# A comparative evaluation of the effects of respiratory diseases on dental caries

**DOI:** 10.1186/s12903-023-03781-7

**Published:** 2024-01-03

**Authors:** Merve Yildirim Ucuncu, Nursen Topcuoglu, Guven Kulekci, Musa Kazim Ucuncu, Mustafa Erelel, Yasemin Benderli Gokce

**Affiliations:** 1 Institute of Graduate Studies in Health Sciences, Istanbul University, Istanbul, Turkey; 2 Faculty of Dentistry, Deparment of Basic Medical Sciences, Istanbul University, Istanbul, Turkey; 3 Faculty of Dentistry, Department of Restorative Dentistry, Altinbas University, Istanbul, Turkey; 4 Faculty of Medicine, Department of Chest Diseases, Istanbul University, Istanbul, Turkey; 5 Faculty of Dentistry, Department of Restorative Dentistry, Istanbul University, Istanbul, Turkey

**Keywords:** Cariogram, Dental Caries, Inhaler Drugs, Oral Bacteria, Saliva

## Abstract

**Purpose:**

The aim of this study is to evaluate the susceptibility of patients suffering from asthma and chronic obstructive pulmonary disease (COPD) to dental caries by analyzing the physical, chemical, and microbiological characteristics of saliva, which are influenced by the medications they use.

**Methods:**

A cohort of 104 individuals, spanning from 18 to 70 years of age, underwent a meticulous categorization based on their unique medical profiles and prescribed medication routines. Subsequently, a comprehensive evaluation was conducted to elucidate potential risk factors associated with dental caries. Alongside the assessment of decayed, missing, and filled teeth (DMFT index), decayed, missing, and filled surfaces (DMFS index), and Green and Vermillion Oral Hygiene Index-Simplified (G&V OHI-S) values, measurements were performed to gauge salivary flow rate, buffering capacity, and the presence of *S. mutans, L. casei, S. aureus*, and *C. albicans*. The acquired data were then inputted into the Cariogram software, enabling the derivation of personalized caries risk profiles for each individual.

**Results:**

The diseased group exhibited significantly elevated levels of DMFT, DMFS, and G&V OHI-S values in comparison to the control group (*p* < 0.01). Moreover, the caries risk levels derived from the Cariogram were found to be significantly higher in patients diagnosed with asthma and COPD (*p* < 0.01). Notably, no substantial distinction was observed between these two experimental groups. Furthermore, it was discerned that COPD patients utilizing two or three distinct medications did not display any discernible variation in terms of their susceptibility to dental caries (*p* > 0.05).

**Conclusion:**

Asthma and COPD patients exhibit an increased susceptibility to dental caries as a result of their medication regimens. Hence, it is highly advisable for these individuals to demonstrate heightened vigilance in terms of oral hygiene practices and seek regular dental check-ups for continuous monitoring and preventive care.

## Introduction

Dental caries represents a complex and dynamic process initiated by bacterial biofilms; this process’s progression is intricately regulated by dietary factors, and it can even be said that several systemic diseases extend an invitation to caries process [1, 2]. Patients using medications for the treatment of asthma and Chronic Obstructive Pulmonary Disease (COPD) may experience adverse effects on their salivary properties. Due to the medications, a shift in the ambient pH towards acidity and reduced salivary volume can facilitate the proliferation of cariogenic microorganisms, which is acknowledged as a contributing factor to the increased risk of dental caries [3, 4].

The worldwide prevalence of asthma exhibits significant variations across different regions. Comparative and standardized methods indicate that the prevalence ranges from 1 to 18% among adults and children [5]. Studies conducted on Turkish populations have reported asthma prevalence rates ranging from 2 to 17% in adults [6, 7]. Moreover, the projections suggest that the prevalence of COPD will experience a rise in the next three decades, with annual deaths attributed to COPD and related causes surpassing 4.5 million by the year 2030 [8]. In the present day, the majority of medications used for asthma and COPD are administered through inhalation, with these inhalers being repeatedly used for extended periods of time. When considering the medications to be employed; in the treatment of asthma, the goal is to reduce airway inflammation to control symptoms, prevent disease progression, and avert exacerbations, achieved through the use of inhaled corticosteroids, beta-2 agonists medications [9]. In the case of COPD, β2-agonists, anticholinergics, inhaled corticosteroids, and their combinations are employed to reduce symptoms, the frequency and severity of exacerbations, and to improve the quality of life [8, 10]. The rising incidence of asthma and COPD, coupled with the increasing number of individuals using inhaler medications, has led to oral health problems associated with these drugs becoming a significant global health issue, not limited to specific populations [11].

The association between oral health issues and asthma, such as dental caries, periodontal problems, erosion, dentofacial anomalies, and changes in the oral mucosa, has been extensively investigated in numerous studies [4, 11–14]. While these studies have examined the oral health of individuals with asthma, the findings have yielded conflicting results [4, 12, 14–16]. It is worth noting that investigations into the relationship between asthma and oral dental health predominantly focus on pediatric patients, with a scarcity of studies conducted on adult individuals [4, 12, 14–17]. Similarly, research exploring the connection between COPD and oral dental health is infrequent in the literature, with only a handful of studies discussing the relationship between COPD and periodontal disease or COPD and caries [18–21].

The objective of this investigation is to compare the physical, chemical, and microbiological traits of saliva, a vital element in promoting oral and dental well-being, in patients with asthma and COPD who employ inhaled medications, with a specific emphasis on assessing their susceptibility to dental caries.

## Materials and methods

### Ethical approval and the calculation of sample size

In the present study, which was performed on 83 adult volunteer individuals, 42 COPD and 41 asthma patients, admitted to the Istanbul University, Faculty of Medicine, Department of Chest Diseasse were determined to be the experimental group. The control group consisted of 21 healthy individuals admitted to the Department of Restorative Dentistry at the Istanbul University (N = 104). This cross-sectional and comparative study was approved by the Istanbul University Faculty of Dentistry Clinical Research Ethics Committee (no: 2017/5). Based on the data from the comprehensive Turkey Oral and Dental Health Profile Research Report was obtained by the State Institution and the Ministry of Health in 2018 [22], revealing the oral and dental health profile of the entire country, the expected average DMFT value for individuals in the healthy group was calculated as µA = 1.7 in our study. In the disease group, the expected average DMFT value was determined as µB = 3. After the variance value was determined to be 1.5, the f value was obtained through the formula. Using the G. Power program with f = 0.41, α = 0.05, and a study power of 0.80 as reference points, it was calculated that the sample size should be at least 21 individuals in each subgroup.

### The inclusion and exclusion criteria

Individuals between the ages of 18 and 70 with at least 10 permanent teeth were included in the present study. The exclusion criteria were using dentures, pregnancy, and the presence of a systemic disease except for asthma and COPD. The control group was selected from individuals in the same age group and without any systemic disease. Experimental groups were selected from asthmatic patients using two drugs (inhaled corticosteroids & β-mimetics), from COPD patients using either two (inhaled corticosteroids and β-mimetics) or three (inhaled corticosteroids, β-mimetic and anticholinergics) pharmaceuticals. Patients using drugs for less than 6 months or longer than 3 years were excluded. All patients were examined by a single clinician (M. Y. U.).

### The evaluation of DMFT/DMFS, G&V OHI-S

The age, gender, type of disease, type of medication, oral hygiene index, dental caries high-risk indicators, and protective factors were recorded. Oral and dental health evaluations were performed by an experienced restorative dentist, using a dental mirror and explorer under a reflector light. DMFT / decayed, missed, and filled surfaces (DMFS) index score and Green and Vermillion Oral Hygiene Index-Simplified (G&V OHI-S) as “excellent” = 0, “satisfactory” = 1, “unsatisfactory” = 2, and “poor” = 3 were measured.

### The calculation of saliva properties and microbial counting

Stimulated saliva samples were gathered for laboratory and culture procedures. Salivary flow rate, buffering capacity, *Streptococcus mutans* (MS), *Lactobacillus casei* (LB), *Staphylococcus aureus* (SA), and *Candida albicans* quantities were calculated. The saliva samples were collected prior to examination. The subjects were instructed to refrain from consuming food, beverages, or brushing their teeth for a minimum of one hour before the sample collection.

#### The calculation of *Streptococcus mutans* quantity

**Fig. 1:**
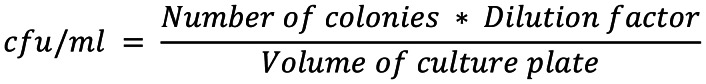
Equation of cfu/ml

Mitis Salivarius Basitrasin Agar (Acumedia Man Inc., Baltimore, Maryland, USA) petri dishes were inoculated with 50 µl of 10^− 3^ and 10^− 4^ diluted samples, which were dispensed onto the surface and spread evenly. Incubation was carried out at 37 °C in a candle extinction jar with an environment containing 5–7% CO_2_ for 48 h [23]. At the end of the incubation period, the blue, opaque, and rough colonies on the Mitis Salivarius Basitrasin Agar surface were counted under magnification. The colony-forming units (cfu/ml) were calculated. Then, this value was then converted to a logarithmic scale to determine the level in saliva (Fig. 1). The *S. mutans* levels were categorized as high (≥ 10^6^ - <10^7^ cfu/ml), moderate (> 10^5^ - <10^6^ cfu/ml), and low (≤ 10^5^ cfu/ml).

#### The calculation of *Lactobacillus casei* quantity

Rogosa SL agar plates (Merck KGaA, Darmstadt, Germany) were inoculated with 50 µl each of 10^− 1^ and 10^− 2^ diluted samples, dispensed onto the surface and spread evenly. Incubation was conducted at 37 °C in a candle extinction jar with an atmosphere containing 5–7% CO_2_ for 48 h [24]. At the end of the incubation period, colonies were counted and calculated in colony-forming units per milliliter. This value was then converted to logarithmic scale to determine the level in saliva (Fig. 1). The *L. casei* levels were categorized as high (≥ 10^5^ cfu/ml), moderate (< 10^4^ – 10^5^ cfu/ml), and low (≤ 10^4^ cfu/ml).

#### The calculation of *Staphylococcus aureus* quantity

0.1 mL aliquots of samples underwent a serial 10-fold dilution process. Subsequently, these diluted samples were plated onto Mannitol Salt Agar (Acumedia Manufacturers Inc, Baltimore, Maryland) and incubated aerobically at 37 °C for a duration of 48 h. Identification of *Staphylococcus aureus* isolates was accomplished through the utilization of the DNase test, which was performed using DNase test agar (Difco Lab, Detroit MI 48,232 − 7058, USA) [25]. The samples with a diagnosis of *S. aureus* were categorized as “carriage” while those without the diagnosis were labeled as “non-carriage”.

#### The calculation of *Candida albicans* quantity

Microbiological aliquots of 0.1 mL samples underwent a serial 10-fold dilution process and were subsequently plated onto Sabouraud Dextrose Agar (Oxoid Ltd, Basingstone, UK) and incubated aerobically at 37 °C for a duration of 48 h. Following this, the identification of *Candida albicans* isolates was accomplished through the performance of the germ tube test [25]. The colony-forming units (cfu/ml) were calculated. This value was then converted to a logarithmic scale to determine the level in saliva (Fig. 1). The level of *C. albicans* was categorized as high (≥ 10^4^ cfu/ml), moderate (> 10^3^–<10^4^ cfu/ml), and low (≤ 10^3^ cfu/ml).

### Cariogram

Using Cariogram software, a profile of each individual’s caries risk was generated. For each individual, the 10 caries-related factors listed below were entered into the Cariogram: Caries history, associated illnesses, food composition, diet intensity, the level of mutans streptococci, the quantity of plaque, the fluoride programme, salivary buffer capacity, salivary release velocity, and medical check-up were assessed. Considering the provided factors, the probability of future caries avoidance was computed. Age-specific DMFT values ​​determined by WHO were used to determine the caries risk. The G&V OHI-S scale was used to assess the degree of plaque accumulation on six teeth (16, 26, 11, 36, 31, and 46). Salivary flow velocity was scored as follows: score 0 = 1 mL/min, score 1 = 0.7-1 mL/min, score 2 ≤ 0.7 mL/min, and score 3 < 0.1 mL/min. According to the criteria of cariogram, dietary habits were documented. The intensity of dietary consumption was also evaluated: 0 = up to a maximum of 3 intakes per day, 1 = up to a maximum of 5 intakes per day, 2 = up to a maximum of 7 intakes per day, and 3 = above 7 intakes per day. Fluoride scores were assigned as 0, 1, 2, and 3 based on the use of fluoridated toothpaste, mouthwash, and varnish. The “clinical judgment” score was given by a single clinician for all individuals. Each participant’s caries risk profile was presented as a pie graph with two colored sections, indicating the “%” likelihood of avoiding caries. If the chance of avoiding caries exceeds 75%, the individual is considered to have a good likelihood of protection against new caries formation, indicating a “low” risk of caries. Conversely, if the chance of avoiding caries is 25% or less, the individual is considered to have a lower likelihood of protection against new caries formation, indicating a “moderate or high” risk of caries [26].

### The statistical analysis

For the purpose of statistical analysis, we utilized the NCSS (Number Cruncher Statistical System) 2007 program, located in the esteemed city of Kaysville, Utah, in the United States of America. Descriptive statistical methods, such as the mean, standard deviation, median, frequency, ratio, minimum, and maximum, were employed to evaluate the data. The quantitative variables, which exhibited normal distribution, were subjected to the Students t test, while the Mann-Whitney U test was employed for the two groups of variables that did not demonstrate normal distribution. In addition, the comparison of three groups with normal distribution was conducted via the one-way ANOVA test, with the Bonferroni test being implemented to detect the specific group responsible for the observed difference. Similarly, the comparison of groups containing three or more variables, which did not exhibit normal distribution, was performed using the Kruskal-Wallis test and Mann-Whitney U test with Bonferroni correction. The qualitative data was compared using the Pearson chi-square test, Fisher’s exact test, and Fisher Freeman-Halton test. In the asthma, COPD, and control groups, it was determined that values such as DMFT, buffering capacity, MS Log_10_, and Cariogram exhibited a normal distribution, while values such as DMFS, G&V OHI-S, the amount of saliva collected, salivary flow rate (ml/min), LB Log_10_, and *Candida albicans* Log_10_ did not follow a normal distribution. Within the COPD group, when comparing two different medication-using subgroups, it was observed that values such as DMFT, buffering capacity, MS Log_10_, and Cariogram demonstrated a normal distribution, whereas values such as DMFS, G&V OHI-S, the amount of saliva collected (5 min), salivary flow rate (ml/min), LB Log_10_, and *Candida albicans* Log_10_ did not exhibit a normal distribution. In order to achieve statistical significance, *p* < 0.05 was employed. The frequency analysis method was utilized to determine the percentage distribution of variables across the groups, and to express these variables in the relevant groups.

## Results

The study was carried out on a total of 104 individuals, 47.1% (n = 49) female and 52.9% (n = 55) male. The individuals enlisted in the research varied in age from 20 to 70 years, with an average age of 54.73 ± 12.64 years. Asthma was detected in 59.2% (n = 29) and COPD in 18.4% (n = 9) of women; while asthma was found in 21.8% (n = 12) and COPD in 60% (n = 33) of men (Table 1). A significant variance was detected between the measurements of DMFT, DMFS, and G&V OHI-S amongst the groups (*p* = 0.001). By the results of the Bonferroni Test, the DMFT value of the patients with asthma and COPD was discovered to be significantly greater than that of the control group. The highest DMFT value was found in patients with COPD. DMFT and DMFS results showed parallelism. G&V OHI-S value was found to be significantly higher in the asthma and COPD groups compared to the control group. The highest G&V OHI-S values ​​were in the asthma group (Table 2). Salivary flow rate (ml/min) and buffering capacity did not show statistically significant differences between the groups (*p* > 0.05), while logarithmically, LB and *Candida albicans* levels were statistically significantly different. The MS Log_10_ level did not display any significant statistical disparity (*p* = 0.1). Conversely, the significant statistical variances were observed in the levels of MS (*p* < 0.05), LB (*p* < 0.001), and *Candida albicans* (*p* < 0.001). The prevalence of *S. aureus* in the control group exceeded that of the experimental group (14.3%). The presence of *S. aureus* eluded the majority of the participants in this present study. No statistical difference was discerned between the groups regarding *S. aureus*, as detailed in Table 3. However, a statistically significant difference was discovered between the experimental and control groups in the assessment of Cariogram. The Cariogram value of the asthma group was found to be lower than that of COPD. No individuals with high caries risk group were found in this study; in addition, the prevalence of individuals with mild risk was determined in the asthma and COPD group (90.2%, 85.7% respectively). While the control group comprised 80% of individuals with a low risk of caries, the experimental groups demonstrated a statistically significant distinction in comparison (Table 4). Moreover, the COPD group was also evaluated in terms of DMFT, DMFS, G&V OHI-S, salivary properties, and microorganism levels according to the number of drug types used in the present study. The results are shown in Table 5.

**Table 1:** Evaluation of Demographic Characteristics

		Groups		
		Asthma (n = 41)	COPD (n = 42)	Control (n = 21)
**Years**	*Min-Max (Median)*	20–69 (55)	27–70 (65,5)	25–65 (48)
	*Mean ± Sd*	51.46 ± 12.39	62.10 ± 9.76	46.38 ± 10.59
		**n (%)**	**n (%)**	**n (%)**
**Sex**	**Female**	29 (59.2)	9 (18.4)	11 (22.4)
	**Male**	12 (21.8)	33 (60.0)	10 (18.2)

**Table 2:** Evaluation of DMFT – DMFS and G&V OHI-S

		Groups			Value	Post Hoc
		^a^Asthma (n = 41)	^b^COPD (n = 42)	^c^Control (n = 21)	*p*	
**DMFT**	*Min-Max (Median)*	4–26 (14)	6–32 (20)	1–19 (7)	F:29.493	**c < a < b**
	*Mean ± Sd*	14.95 ± 5.68	19.14 ± 5.86	7.81 ± 4.39	$^\equiv$ 0.001**	
**DMFS**	*Min-Max (Median)*	4-140 (66)	18–220 (94)	3–60 (22)	χ^2^:40.061	**c < a < b**
	*Mean ± Sd*	61.61 ± 34.2	87.62 ± 36.82	23.38 ± 17.57	^*∆*^ 0.001**	
**G&V OHI-S**	*Min-Max (Median)*	0.33–2.33 (1)	0.33-2 (1)	0–0,5 (0,33)	χ^2^:44.167	**c < a;b**
	*Mean ± Sd*	0.95 ± 0.42	0.92 ± 0.43	0.26 ± 0.15	^*∆*^ 0.001**	

^*∆*^*Kruskal Wallis Test*$^\equiv$*Oneway Anova* **p < *0.01*

**Table 3:** Evaluation of Saliva Flow Rates and Buffering Capacity, Presence of MS, LB, and *Candida* and *S. Aureus*

		Groups			Value	Post Hoc
		^a^Asthma (n = 41)	^b^COPD (n = 42)	^c^Control (n = 21)	*p*	
**The amount of saliva collected (5 min)**	*Min-Max (Median)* *Mean ± Sd*	0.5–8.5 (5.5) 4.95 ± 2.14	1–8 (6) 5.31 ± 1.96	3.5–10 (6) 6.14 ± 1.78	χ^2^:3.332 ^*∆*^ ***0.189***	**-**
**Salivary flow rate (ml/min)**	*Min-Max (Median)* *Mean ± Sd*	0.1–1.7 (1.2) 0.99 ± 0.43	0.2–1.6 (1.2) 1.06 ± 0.39	0.7-2 (1.2) 1.23 ± 0.36	χ^2^:3.182 ^*∆*^ ***0.204***	**-**
**Buffering capacity**	*Min-Max (Median)* *Mean ± Sd*	4-6.5 (5.5) 5.44 ± 0.58	4.5–6.5(5.75) 5.52 ± 0.57	4.5-6 (5) 5.45 ± 0.55	F:0.250 $^\equiv$ ***0.779***	**-**
**MS Log**_**10**_	*Min-Max (Median)*	3.95–7.48 (6.51)	3.95-8 (5.9)	3.95–7.04 (5.6)	F:2.355	**-**
	*Mean ± Sd*	6.2 ± 0.9	6.02 ± 1.09	5.64 ± 0.72	$^\equiv$ ***0.100***	
**Level of MS** *n(%)*	**Low**	2 (4.9)	7 (16.7)	2 (9.5)	χ^2^:9.656	**-**
	**Moderate**	15 (36.6)	15 (35.7)	14 (66.7)	^*∩*^ 0.042*	**a;b < c**
	**High**	24 (58.5)	20 (47.6)	5 (23.8)		**c < a**
**LB Log**_**10**_	*Min-Max (Median)*	1.95–5.96 (4.78)	1.95–6.6 (5)	2.95–5.45 (4.48)	χ^2^:15.049	**c < b**
	*Mean ± Sd*	4.65 ± 0.69	4.97 ± 0.86	4.37 ± 0.58	^*∆*^ 0.001**	
**Level of LB** *n(%)*	**Low**	4 (9.8)	4 (9.5)	10 (47.6)	χ^2^:20.114	**a;b < c**
	**Moderate**	21 (51.2)	16 (38.1)	8 (38.1)	^*∏*^ 0.001**	**-**
	**High**	16 (39.0)	22 (52.4)	3 (14.3)		**c < a;b**
***Candida albicans*** ** Log**_**10**_	*Min-Max (Median)*	0.95–4.9 (3.2)	0.95–4.9 (3.24)	0.95–3.82 (0.95)	χ^2^:14.482	
	*Mean ± Sd*	2.71 ± 1.36	2.63 ± 1.49	1.35 ± 0.88	^*∆*^ 0.001**	**c < a;b**
**Level of* Candida albicans*** n*(%)*	**Low**	17 (41.5)	20 (47.6)	19 (90.5)	χ^2^:19.893	**a;b < c**
	**Moderate**	17 (41.5)	9 (21.4)	2 (9.5)	^*∏*^ 0.001**	**b;c < a**
	**High**	7 (17.0)	13 (31.0)	0 (0.0)		**c < b**
***S. aureus*** *n(%)*	**Non-carriage**	38 (92.7)	39 (92.9)	18 (85.7)	χ^2^:1.201	**-**
	**Carriage**	3 (7.3)	3 (7.1)	3 (14.3)	^*∩*^ ***0.600***	

^*∆*^ *Kruskal Wallis Test* ^*∏*^ *Pearson Chi-Square Test* $^\equiv$ *Oneway Anova*

^*∩*^*Fisher Freeman Halton Test* *p < 0,05 **p < *0,001*

**Table 4:** Evaluation of Cariogram Measurements and Risk Levels

		Group			Value	Post Hoc
		^a^Asthma (n = 41)	^b^COPD (n = 42)	^c^Control (n = 21)	*p*	
**Cariogram**	*Min-Max (Median)*	23–77 (49)	32–77 (51)	54–87 (71)	F:28.680	**a;b < c**
	*Mean ± Sd*	48.44 ± 11.54	52.19 ± 11.09	70.1 ± 9.13	$^\equiv$ 0.001**	
**Risk** * n(%)*	**Low**	4 (9.8)	6 (14.3)	17 (81.0)	χ^2^:41.618	
	**Mild**	37 (90.2)	36 (85.7)	4 (19.0)	^*∏*^ 0.001**	**c < a;b**

^*∏*^*Pearson Chi-Square Test*$^\equiv$*Oneway Anova*. **p < *0,01*

**Table 5:** Evaluations in terms of the number of drug types in COPD group

The group of COPD		The number of using drug			Value
		Two drugs (n = 21)		Three drugs (n = 22)	*p*
**DMFT**	*Min-Max (Median)*	10–28 (20)		6–32 (21)	t:-0.627
	*Mean ± Sd*	18.57 ± 5.67		19.71 ± 6.13	^*⌂*^ ***0.534***
**DMFS**	*Min-Max (Median)*	18–130 (96)		26–220 (88)	Z:-0.214
	*Mean ± Sd*	86.1 ± 29.66		89.14 ± 43.53	^*∞*^ ***0.831***
**G&V OHI-S**	*Min-Max (Median)*	0.33-2 (0.75)		0.4-2 (1)	Z:-0.655
	*Mean ± Sd*	0.87 ± 0.43		0.96 ± 0.44	^*∞*^ ***0.513***
**The amount of saliva collected (5 min)**	*Min-Max (Median)*	2–8 (6)		1–8 (5)	Z:-0.215
	*Mean ± Sd*	5.43 ± 1,85		5.19 ± 2.11	^*∞*^ ***0.830***
**Salivary flow rate (ml/min)**	*Min-Max (Median)*	0.4–1.6 (1.2)		0.2–1.6 (1)	Z:-0.215
	*Mean ± Sd*	1.09 ± 0.37		1.04 ± 0.42	^*∞*^ ***0.830***
**Buffering Capacity**	*Min-Max (Median)*	4,5–6 (5,5)		4.5–6.5 (6)	t:0.266
	*Mean ± Sd*	5.55 ± 0.52		5.5 ± 0.63	^*⌂*^ ***0.792***
**MS Log**_**10**_	*Min-Max (Median)*	3.95-8 (5.9)		3.95–7.9 (6)	t:0.639
	*Mean ± Sd*	6.13 ± 0.99		5.91 ± 1.19	^*⌂*^ ***0.526***
**Levels of MS** n(%)	**Low**	3 (14.3)		4 (19.0)	χ^2^:0.998
	**Moderate**		9 (42.9)	6 (28.6)	^*∩*^ ***0.702***
	**High**		9 (42.9)	11 (52.4)	
**LB Log**_**10**_	*Min-Max (Median)*	4.3–6.6 (5)		1.95–6.15 (5)	Z:-2.018
	*Mean ± Sd*	5.3 ± 0.62		4.64 ± 0.96	^*∞*^ 0.044*
**Levels of LB** n(%)	**Low**	0 (0.0)		4 (19.0)	χ^2^:4.698
	**Moderate**	10 (47.6)		6 (28.6)	^*∩*^ ***0.090***
	**High**	11 (52.4)		11 (52.4)	
***Candida albicans*** **Log**_**10**_	*Min-Max (Median)*	0.95–4.6 (2.3)		0.95–4.9 (3.6)	Z:-0.679
	*Mean ± Sd*	2.45 ± 1.46		2.81 ± 1.53	^*∞*^ ***0.497***
**Levels of** *** Candida albicans***	**Low**	12 (57.1)		8 (38.1)	χ^2^:1.620
	**Moderate**	4 (19.0)		5 (23.8)	^*∩*^ ***0.531***
	**High**	5 (23.8)		8 (38.1)	
***S. aureus*** *n(%)*	**Non-carriage**	20 (95.2)		19 (90.5)	χ^2^:0.359
	**Carriage**	1 (4.8)		2 (9.5)	^*↕*^ ***1.000***
**Cariogram**	*Min-Max (Median)*	32–75 (53)		35–77 (49)	t:-0.691
	*Mean ± Sd*	53.38 ± 11.49		51 ± 10.83	^*⌂*^ ***0.493***
**Risk** * n(%)*	**Low**	3 (14.3)		3 (14.3)	χ^2^:0.000
	**Mild**	18 (85.7)		18 (85.7)	^*↕*^ ***1.000***
**The number of Attack** * n(%)*	**No attack**	18 (85.7)		18 (85.7)	χ^2^:0.825
	**1 attack**	1 (4.8)		2 (9.5)	^*∩*^ ***1.000***
	**≥ 2 attacks**	2 (9.5)		1 (4.8)	

^*∩*^ *Fisher Freeman Halton Test* ^*⌂*^ *Student-t Test* ^*∞*^ *Mann Whitney U Test*

^*↕*^*Fisher’s Exact Test* *p < 0,05 **p < *0,01*

## Discussion

Oral and dental health are integral aspects of overall well-being. The correlation between oral health and general health has been firmly established, as highlighted in the Oral Health Report published by the American General Health Association in 2000 [27]. In light of the aforementioned information, considerably the respiratory diseases, numerous investigations examining the intraoral findings of individuals with asthma have yielded conflicting results regarding the prevalence of caries. Some studies suggest a higher risk of caries among asthma patients [4, 12, 14, 28–31], while others report no significant association between asthma and caries [15, 16]. However, it is important to note that most research on the relationship between asthma and oral health has predominantly focused on pediatric patients [2, 4, 14–16, 28, 32], with a relatively limited number of studies conducted on adult individuals [17, 32]. In the literature, there is a scarcity of research exploring the correlation between COPD and oral dental well-being. The relationship between COPD and periodontal disease [18, 19, 33, 34] as well as COPD and caries has been addressed in only a few studies [20, 21, 34, 35].

Considering the utilization of inhaled medications such as corticosteroids and ß-mimetics by individuals with asthma and COPD, both experimental groups were assessed. Within the COPD group, two subgroups were identified, one involving the subclinical use of these two medications and the other exploring the administration of inhaled anticholinergic drugs. The use of inhaler corticosteroids in asthma and COPD patients has been suggested to render the oral environment more susceptible to cariogenic microorganisms. Moreover, numerous studies have indicated that inhaled ß-mimetics and anticholinergics can contribute to an increased risk of dental caries due to the resulting dry mouth. Consequently, researchers have incorporated inhaled corticosteroids and ß-mimetic drugs in their investigations of the oral manifestations in patients with asthma and COPD [14, 20, 21, 36] The primary objective of this study was to assess the utilization of inhaled medications among adult individuals with asthma and COPD and investigate the impact of saliva on the physical, chemical, and microbiological properties of these medications. Furthermore, the influence of this interaction on caries activity was examined and comparatively evaluated.

In this study, the participants with asthma and COPD exhibited significantly higher DMFT values compared to the control group. This conclusion is supported by numerous studies [3, 17, 21, 29, 31, 35, 37–41]. However, the study conducted by Paganini et al. [36] and Świątkowska-Bury et al. [2] did not find a significant difference in DMFT between the control and experimental groups. The DMFS values of both asthmatic and COPD patients were markedly higher than those of the control groups, a finding that is consistent with the majority of studies on this subject [14, 17, 29, 31, 42, 43]. This study focused on the oral condition of asthma and COPD patients using inhaled corticosteroids, while Ryberg et al. [12] specifically examined individuals using inhaled ß-mimetics and found no statistically significant differences in DMFS values between the experimental and control groups. The use of inhalers in the treatment of asthma and COPD may increase the susceptibility to caries due to the presence of lactose as a carrier in their contents. Furthermore, inhaled ß-mimetic agents and anticholinergics are believed to have a drying effect on the mouth, while inhaled corticosteroids, with their oral immunosuppressive effects, create an environment conducive to the development of caries. Therefore, it can be postulated that the increased DMFT and DMFS values observed in the experimental groups in this study are the result of the oral effects of inhaled drugs.

In present study, the oral hygiene index revealed that both asthmatic and COPD patients had significantly higher amounts of dental plaque compared to the control group. This finding was supported by related studies conducted by Raj et al. and Botelho et al., where the experimental groups exhibited greater plaque accumulation [21, 38]. The use of inhaled medications often leads to an unpleasant taste in the mouth, which may prompt individuals to consume more snacks and sugary foods. Consequently, an increase in dental plaque accumulation can be observed in the experimental groups. In contrast, Stenson et al. found no difference in dental plaque levels between asthmatic and healthy patients [43]. They attributed this finding to the implementation of oral hygiene education programs targeted at adolescents with asthma in their country [43]. Similarly, Khijmatgar et al. did not identify a statistically significant difference in dental plaque amounts between COPD patients and controls [35]. They attributed this to the patients cleaning their oral cavity after each use of an inhaler. No significant differences in salivary flow rates were observed between the experimental groups and the control group in our study. These findings diverge from the prevailing evidence reported in previous studies [12, 14, 20, 36, 40, 43, 44]. This disparity may be attributed to variations in parameters such as age, frequency of daily drug use, duration of use, and additional medication usage across different studies. When considering the buffering capacities of saliva, no significant differences were observed among the groups investigated in this study. Similar findings have been reported in previous studies [12, 14, 36, 43]. However, Mazzoleni et al. conducted a study specifically focusing on individuals with asthma using short-acting ß-mimetic drugs, which yielded contrasting results [3]. It can be hypothesized that the lower buffering capacity may be attributed to the frequent use of short-acting drugs throughout the day.

Some researchers argue that the use of inhaler medications leads to an increase in the number of mutans streptococci [3, 12, 38], while others have not found any significant differences [14, 43]. In the context of this study, no statistically significant differences were observed between the experimental groups and the control group in terms of the quantity of mutans streptococci present in saliva. It is believed that this discrepancy may be attributed to variations in the duration and frequency of inhaler use among the individuals participating in the trials. In terms of the number of lactobacilli in saliva, no statistically significant difference was found between the control group and the asthma group in this study. These results align with previous studies [12, 38, 41, 43]. However, the number of lactobacilli in saliva was significantly higher in individuals with COPD compared to both the asthma experimental and control group. Inhaled corticosteroids, ß-mimetic drugs, and anticholinergics, particularly dry powder inhaled medications, may contribute to oral immunosuppression, dry mouth, and increased salivary viscosity. The lactose content used as a carrier agent in dry powder inhaled medications may explain the higher number of lactobacilli in individuals with COPD compared to the control group. Whilst individuals in the asthma group of our study were prescribed two types of inhalers, those in the COPD group were prescribed either two or three different types of medications. The increased number of medications and daily dosage intensify the intraoral effects. Therefore, individuals with COPD may exhibit higher lactobacilli values than those with asthma. The number of yeasts present in the saliva of individuals with asthma and COPD in the experimental groups of this study was significantly higher compared to the control group. This finding is consistent with other studies conducted on this topic [21, 35]. The carriage rate of *S. aureus* was found to be 7.14% in individuals with asthma and COPD, while it was 14.28% in the control group. It is well-known that *S. aureus* can inhabit both supragingival and subgingival plaque in humans [45–48]. This microorganism has been associated with infections related to implants and other biomaterials [49, 50], and therefore, it has been considered a potential contributor to the development of various oral diseases [47]. Numerous studies have provided evidence supporting the concept of oral microbial dysbiosis, which refers to the disruption of the oral microbial community, as well as alterations in the oral microenvironment, including the progression of periodontitis and denture stomatitis, and the use of dental prostheses, all of which can promote the colonization of *S. aureus* in the oral cavity [51–54]. These findings help explain why the use of dental prostheses can lead to an increase in the number of *S. aureus* in the oral cavity [55]. Dental prostheses have also been considered potential reservoirs for respiratory pathogens [56, 57]. Within the scope of this study, no association was found between asthma, COPD, and selective oral carriage of *S. aureus*. However, it is important to note that the presence of *S. aureus* in the oral cavity may pose a risk for respiratory infections, thereby impacting the prognosis of asthma and COPD.

The multifactorial etiology of dental caries necessitates the need for a caries risk assessment model that encompasses various contributing factors. Through the computer software called Cariogram, the risk of dental caries in individuals is determined by quantifying and combining various etiological factors using algorithmic calculations. Each etiological factor is evaluated and weighted to calculate the percentage of risk [58]. The caries risk categories can range from 0 to 100%: in children, 0–20% represents high risk, 21–80% (21–40%, 41–60%, 61–80%) moderate risk, and 81–100% low risk [26]. In adults, as in our study, values of 75% and above indicate a low risk of developing caries, while individuals with values below 25% are considered to have a moderate-high risk and a moderate-high probability of developing caries [58]. Studies evaluating the caries risk in individuals with asthma and COPD using the Cariogram model are scarce in the literature [43]. However, the obtained results are consistent with our study’s findings. Assessing caries risk can be challenging due to the multiple factors influencing its development. To successfully determine caries risk, socioeconomic factors and other factors that can impact caries risk should be considered. Although one limitation of the Cariogram model is the neglect of socioeconomic factors and age; affordability, user-friendly interface, accessibility, and comprehensibility make it a valuable tool for dentists in implementing preventive measures for individuals. Furthermore, the Cariogram has been found to be successful in predicting caries risk and has been utilized in our study for these reasons [59, 60].

Individuals with COPD were categorized into two subgroups based on their medication type, and the factors associated with caries were compared in the present study. There were no statistically significant differences observed in terms of DMFT and DMFS values, dental plaque amounts (OHI), salivary flow rate and buffering capacities, levels of mutans streptococci and lactobacilli in saliva, yeast count, and caries risks according to the cariogram analysis among individuals with COPD based on their medication type. Both individuals with COPD and asthma are more prone to dental caries due to the oral immunosuppressive effects of inhaled corticosteroids and the drying effects of ß-mimetics on the oral cavity. The use of a combination of three medications did not lead to a significant alteration in caries risk. Long-acting anticholinergic drugs are typically administered once a day, as they provide 24-hour effectiveness. It is believed that taking an additional dose of an inhaler per day does not result in a significant change in the oral environment.

## Conclusion

Our research findings were carefully analyzed in conjunction with the results of previous studies, revealing that the use of inhaled medications poses a significant risk factor for dental caries. Individuals who rely on these medications are found to have a substantially higher risk of developing dental caries compared to the general population. To mitigate oral health issues in patients diagnosed with asthma and COPD, it is crucial for healthcare professionals to identify these individuals as high-risk candidates for dental caries and implement personalized preventive treatments. When prescribing inhaled medications to newly diagnosed patients, respiratory physicians should collaborate with dentists to ensure comprehensive care. By adopting a multidisciplinary approach, it is possible to prevent asthma and COPD patients from experiencing adverse oral effects associated with inhaled medications, ultimately enhancing their overall quality of life.

## Data Availability

The datasets used and/or analysed during the current study available from the corresponding author on reasonable request.
